# Characterization of internalin genes in *Listeria monocytogenes* from food and humans, and their association with the invasion of Caco-2 cells

**DOI:** 10.1186/s13099-019-0307-8

**Published:** 2019-06-10

**Authors:** Xudong Su, Guojie Cao, Jianmin Zhang, Haijian Pan, Daofeng Zhang, Dai Kuang, Xiaowei Yang, Xuebin Xu, Xianming Shi, Jianghong Meng

**Affiliations:** 1 Department of Food Science and Technology, MOST-USDA Joint Research Center for Food Safety, School of Agriculture and Biology, State Key Lab of Microbial Metabolism, Shanghai Jiao Tong University, Shanghai, 200240 China; 2 Department of Nutrition & Food Science, University of Maryland, College Park, MD 20742 USA; 3 Shanghai Municipal Center for Disease Control and Prevention, Shanghai, 200336 China

**Keywords:** *L. monocytogenes*, Internalins, *inlA*, PMSC mutation, Invasion

## Abstract

**Background:**

Internalins are surface proteins that are utilized by *Listeria monocytogenes* to facilitate its invasion into human intestinal epithelial cells. The expression of a full-length InlA is one of essential virulence factors for *L. monocytogenes* to cross the intestinal barrier in order to invade epithelial cells.

**Results:**

In this study, the gene sequences of inlA in 120 *L. monocytogenes* isolates from food (n = 107) and humans (n = 13) were analyzed. Premature stop codon (PMSC) mutations in *inlA* were identified in 51 isolates (50 from food and 1 from human). Six mutation types of PMSCs were identified. Among the 51 isolates with PMSCs in *inlA*, there were 44 serogroup 1/2c, 3c isolates from food, of which seven belonged to serogroups 1/2a, 3a. A total of 153,382 SNPs in 2247 core genes from 42 genomes were identified and used to construct a phylogenetic tree. Serotype 1/2c isolates with inlA PMSC mutations were grouped together. Cell culture studies on 21 isolates showed that the invasion to Caco-2 cells was significantly reduced among isolates with inlA PMSC mutations compared to those without PMSC mutations (P < 0.01). The PMSC mutations in *inlA* correlated with the inability of the *L. monocytogenes* isolates to invade Caco-2 cells (Pearson’s coefficient 0.927, *P *< 0.01).

**Conclusion:**

Overall, the study has revealed the reduced ability of *L. monocytogenes* to invade human intestinal epithelial cells in vitro was linked to the presence of PMSC mutations in *inlA*. Isolates with PMSC mutations shared the same genomic characteristics indicating the genetic basis on the potential virulence of *L. monocytogenes* invasion.

**Electronic supplementary material:**

The online version of this article (10.1186/s13099-019-0307-8) contains supplementary material, which is available to authorized users.

## Introduction

*Listeria monocytogenes* is a foodborne pathogen that can cause gastroenteritis in healthy individuals, meningitis in immunocompromised individuals, and abortions in pregnant women. It has a high case fatality rate of 20–30% [9]. Many types of foods, including meat, poultry, dairy, and vegetable products have been implicated as transmission vehicles for *L. monocytoge*nes [1, 44, 63]. In view of the foodborne route of transmission of *L. monocytogenes* and the potential severity of listeriosis, strict food safety regulations have been established, including “zero tolerance” approach for ready-to-eat food in the United States [4].

*Listeria monocytogenes* possesses an array of virulence factors, which allow it to infect, survive, and replicate in a variety of host cell types. Numerous studies have been conducted to investigate the adhesion, invasion, and/or virulence regulation of *L. monocytogenes.* The roles of virulence factors (i.e., PrfA, ActA, InlA, InlB, InlC, LAP, Ami, p60, Auto) in *L. monocytogenes* pathogenesis have been well characterized in different cell types or animal models [2, 5, 9, 12, 13, 25, 33, 42, 53, 56]. Surface proteins of *L. monocytogenes* associate with a variety of different cell surface structures. Internalin surface molecules harbor amino acid leucine-rich repeats that interact with specific host-receptors to promote the invasion of *L. monocytogenes* into selected host cells [3]. InlA identified as a virulence factor in 1991 [18] is a covalently linked bacterial cell-wall protein that binds to E-cadherin on the host epithelial cell, and is associated with crossing the intestinal barrier [9, 38, 40]. Entry into host cells by bacteria has been investigated in great detail. The interaction of internalin with its receptor, E-cad, has been the focus of several studies [35, 46, 47]. InlA has been used as a virulence marker for detecting *L. monocytogenes* in food [8, 23, 28, 41, 50].

*Listeria monocytogenes* carrying premature stop codon (PMSC) mutations in *inlA* produce a truncated form of InlA that is secreted rather than anchored to the bacterial cell wall [16, 20, 37]. A total of 21 mutation types of *inlA* have been identified [19, 20, 58]. The strains with truncated InlA were virulence-attenuated in a guinea pig model [36, 59], and exhibited attenuated invasion of Caco-2 human intestinal epithelial cells [11, 15]. *L. monocytogenes* is a diverse species with a structured population that includes at least three phylogenetic lineages, and two lineages (I and II) are common [43]. Serotypes 1/2b and 4b belong to lineage I, whereas serotypes 1/2a and 1/2c belong to lineage II. Lineage II strains carry *inlA* PMSC mutations more frequently than lineage I strains [39, 48]. However, PMSC in *inlA* have also be identified in isolates from human patients, indicating their ability in causing listeriosis [22, 37]. Additional genomics data would provide useful information to determine potential virulence of *L. monocytogenes* with PMSC mutations.

Several studies on *L. monocytogenes* conducted in China focused on the distribution, molecular subtyping and antimicrobial susceptibility of the pathogen recovered from ready-to-eat (RTE) food products [7, 60, 61]. PMSCs in *inlA* and characteristics of *L. monocytogenes* genomes have not been reported. The aim of the present study was to characterize internalin gene and their association with the ability to invade host cells, and to conduct phylogenetic analysis of selected genomes of *L. monocytogenes* recovered from both food and humans in Shanghai, China.

## Materials and methods

### Bacterial strains

A total of 120 *L. monocytogenes* isolates recovered from 2004 to 2013 in Shanghai, China, were examined in the study, including 30 isolates from duck, 28 from chicken, 27 from beef, 18 from pork, 2 from vegetables, 1 from fish, and 1 from yogurt as well as 13 clinical isolates that were collected from patients admitted to three hospitals in Shanghai. The clinical isolates were recovered from 11 blood and two cerebrospinal fluid (CSF) specimens. All isolates had previously been characterized by serogroup and lineage typing [52]. *Listeria innocua* ATCC33090, *L. monocytogenes* EGD-e and *L. monocytogenes* serotype 4b strain 4bG were used as reference strains for Caco-2 cell invasion assay.

### DNA sequencing

The sequencing of *inlA* gene was performed to determine *inlA* mutation types. Mutation types 1–18 have been summarized previously by Van Stelten et al. [58]. Previously published DNA primer sets were used for PCR amplification and sequencing of *inlA* [39]. PCR products were sequenced at Shenggong Inc, Shanghai, China. The *inlA* sequences of the 120 *L. monocytogenes* isolates were assembled using Seqman software (DNASTAR, Lasergene), and aligned using the CLUSTAL W program (MEGALIGN, DNASTAR, Lasergene). A phylogenetic tree was constructed using the maximum likelihood method [55]. DNAMAN *v.*6.0 software (Lynnon Biosoft, Qc, Canada) was used for the translation of DNA into protein.

### Whole genome sequencing analysis

Whole genome sequencing was performed on four *L. monocytogenes* isolates, including three food isolates carrying PMSC mutations in *inlA* and one human isolate with full-length *inlA*, using Illumina MiSeq (Illumina, San Diego, CA) with MiSeq Reagent Kit v2 (500 cycle), Nextera XT DNA Sample Preparation kit, and Nextera XT Index Kit. Genomics data were annotated using the NCBI Prokaryotic Genomes Annotation Pipeline. All *L. monocytogenes* genomes were annotated at NCBI [51].

### Phylogenetic relationship of *L. monocytogenes* genomes

Phylogenetic analysis of 42 genomes was performed (Table 1), including four genomes from the current study, 17 genomes from our pervious study [62] and 21 publicly available genomes. The deduced amino acid sequences of all CDSs from the 42 genomes were adjusted to a prescribed format and were grouped into homologous clusters using OrthoMCL version 2.0.9 [29] based on sequence similarity. The BLAST reciprocal best hit algorithm [34] was employed with a 70% match cutoff and 1e−5 e-value cutoff, and Markov Cluster Algorithms (MCL) [14] were applied with an inflation index of 1.5. For each orthologous cluster, protein sequences were aligned using Clustal Omega version 1.2.1 [49]. Single nucleotide polymorphisms (SNPs) were identified from core genes in the genome. To reconstruct evolutionary relatedness, a phylogenetic tree was constructed using the maximum likelihood method [55].

**Table 1:** Origin and genetic characteristics of 42 *L. monocytogenes* isolates

Strain	Serotype/lineage	Genome size (Mb)	GC content (%)	Source	Year	Country	GenBank accession
SHL001	1/2a, II	2.95	37.9	CSF^c^	2007	China	APIB00000000^b^
SHL002	1/2b, I	3.12	37.9	Blood	2007	China	APIC00000000 ^b^
SHL003	1/2b, I	3.00	37.9	Blood	2008	China	LVYC00000000
SHL004	1/2a, II	3.01	37.8	Blood	2008	China	APID00000000^b^
SHL005	1/2a, II	2.88	37.9	Blood	2008	China	APIE00000000^b^
SHL006^a^	1/2c, II	2.93	37.9	Blood	2010	China	APIF00000000^b^
SHL007	1/2b, I	2.98	37.9	Blood	2011	China	APIG00000000^b^
SHL008	1/2b, I	3.01	37.9	Blood	2012	China	APIH00000000^b^
SHL009	1/2a, II	2.87	37.9	Blood^d^	2012	China	APII00000000^b^
SHL010	1/2b/I	3.08	37.9	Blood	2012	China	APIJ00000000^b^
SHL011	1/2a, II	2.87	37.9	Blood	2011	China	APIK00000000^b^
SHL012	1/2b, I	2.93	37.9	CSF^c^	2010	China	APIL00000000^b^
SHL013	1/2a, II	2.86	37.9	Blood^d^	2012	China	APIM00000000^b^
LM430^a^	1/2c, II	2.95	37.9	Pork	2008	China	AWWQ00000000^b^
LM438	1/2a, II	2.96	38.7	Beef	2008	China	AWWR00000000^b^
LM440^a^	1/2c, II	2.97	37.7	Fish	2008	China	AWWS00000000^b^
LM469	1/2a, II	2.95	38.7	Bean	2004	China	AWWT00000000^b^
LM470^a^	1/2c, II	2.94	37.8	Vegetable	2004	China	AWWU00000000^b^
SHL12-2^a^	1/2c, II	2.99	37.9	Pork	2012	China	LRTW00000000
SHL12-22^a^	1/2a, II	2.97	37.9	Duck	2012	China	LRTX00000000
SHL13-12^a^	1/2a, II	2.98	37.9	Duck	2013	China	LRTY00000000
EGD-e	1/2a, II	2.94	38.0	Rabbit	1926	UK	AL591824.1
10403S	1/2a, II	2.90	38.0	Human	1968	U.S.	CP002002
F6900	1/2a, II	2.97	37.7	Human	1989	U.S.	AARU02000000
J2818	1/2a, II	2.97	37.7	Human	2000	U.S.	AARX02000000
J0161	1/2a, II	3.00	37.9	Human	2000	U.S.	CP002001
08-5578	1/2a, II	3.03	38.0	Human	2008	Canada	CP001602.1
08-5923	1/2a, II	3.00	38.0	Human	2008	Canada	CP001604
SLCC5850	1/2a, II	2.91	38.0	Rabbit	1924	UK	FR733647
F6854	1/2a, II	2.95	37.8	Hot dog	1988	U.S.	AADQ01000000
C1-387	1/2a, II	2.99	37.9	Food	1999	U.S.	CP006591
FSL N3-165	1/2a, II	2.88	37.8	Soil	N.A.	U.S.	AARQ02000000
SLCC2755	1/2b, I	2.97	38.1	Human	N.A.	N.A.	NC_018587
FSL J1-194	1/2b, I	2.99	37.8	Human	N.A.	U.S.	AARJ00000000
R2-502	1/2b, I	3.03	37.9	Food	1994	U.S.	CP006594
FSL R2-503	1/2b, I	2.99	37.8	Human	1994	U.S.	AARR00000000
N1-011A	1/2b, I	3.01	37.9	Environment	N.A.	U.S.	CP006597
J1-108	4b, I	2.98	38.0	N.A.	2013	U.S.	NC_021825.1
F2365	4b, I	2.90	37.9	Human	1985	U.S.	AE017262.2
FSL R2-561	1/2c, II	2.97	38.0	N.A.	N.A.	N.A.	NC_017546
SLCC 2372	1/2c, II	2.97	38.0	N.A.	N.A.	N.A.	NC_018588
J2-031	1/2c, II	2.96	37.9	Human	1996	U.S.	CP006593

^a^Isolate with a premature stop codon (PMSC) mutation in *inlA*

^b^Isolate whose whole genome was sequenced in a previous study [62]

^c^Cerebrospinal fluid

^d^Caused human death

### Invasion assays

Invasion assays on 21 *L. monocytogenes* isolates were performed using Caco-2 (ATCC HTB-37) cells as previously described [10, 54]. Briefly, the cells were harvested from confluent cell cultures and suspended at a concentration of 1 × 10^5^ cells/mL in Dulbecco’s modified Eagle’s medium (DMEM) containing 10% fetal bovine serum and 1% non-essential amino acids. A 24-well tissue culture plate was seeded with 1 mL per well to confluence for 48 h at a final density of approximately 3.5 × 10^5^ cells per well. The invasion assays were performed by incubating *L. monocytogenes* with Caco-2 epithelial cells at a ratio of 100:1 [10, 54]. The viable count was determined retrospectively by culturing tenfold serial dilution in PBS onto freshly prepared brain heart infusion (BHI) agar plates. *L. monocytogenes* and epithelial cells were co-incubated for 1 h at 37 °C under 5% CO_2_ air atmosphere. To determine the number of the bacterium that had been internalized into epithelial cells, 90 min incubation in DMEM medium containing 150 μg/mL gentamicin (Sigma, St. Louis, Missouri, USA) was performed in each well to kill extracellular bacterial cells. After washing three times with PBS, epithelial cells were then lysed to release the intracellular bacterial cells. The number of *L. monocytogenes* that had invaded the cells was determined by plating serial dilutions of the suspensions onto BHI agar plates. The detection limit of the cell invasion assay was 33 CFU/mL. Blank wells were used as negative controls, and each assay was performed in triplicate and replicated three times. *L. innocua* ATCC33090, and *L. monocytogenes* strains EGD-e and 4bG were included in the invasion assay as reference strains. Data from three replicates were included in statistical analysis using SAS software 9.1 (SAS Institute Inc., Cary, NC, USA) to determine differences between bacterial count means among the different isolates. *P*-values of < 0.05 were considered as statistically significant.

## Results

### Mutation genotype in inlA of *L. monocytogenes*

The *inlA* gene was successfully amplified in the 120 *L. monocytogenes* isolates from food and humans. Mutation types 4, 6, 8, 11, 12 and 19 were identified among 51 of the 120 isolates (Table 2, Additional file 1: Table S1). Forty-three food isolates and one human isolate belonged to serogroup 1/2c, 3c. Seven food isolates belonged to serogroups 1/2a and 3a. Serogroup 1/2b and 3b isolates possessed no PMSC mutations in their *inlA* genes. PMSC mutation type 19 was identified in 10 foods isolates, which expressed a truncated InlA protein (325aa length). Human isolate SHL013 (serotype 1/2a) had 9 nucleotide deletions in *inlA*. Human isolate SHL006 carried mutation type 12 in *inlA*.

**Table 2:** Distribution of PMSC mutations and codon deletions in *inlA* of 51 isolates among 120 *L. monocytogenes* obtained from food and human clinical samples

Serogroup	InlA PMSC								Codon deletion
	Food isolates	Human isolates	Type 4	Type 6	Type 8	Type 11	Type 12	Type 19	
1/2a, 3a	7/38 (18.4%)	0/6 (0.0%)	2	5	0	0	0	0	1
1/2b, 3b, 7	0/23 (0.0%)	0/6 (0.0%)	0	0	0	0	0	0	0
1/2c, 3c	43/46 (93.5%)	1/1 (100%)	18	0	6	6	4	10	0
Total	50	1	20	5	6	6	4	10	1

### Phylogenetic analysis of inlA

**Fig. 1:**
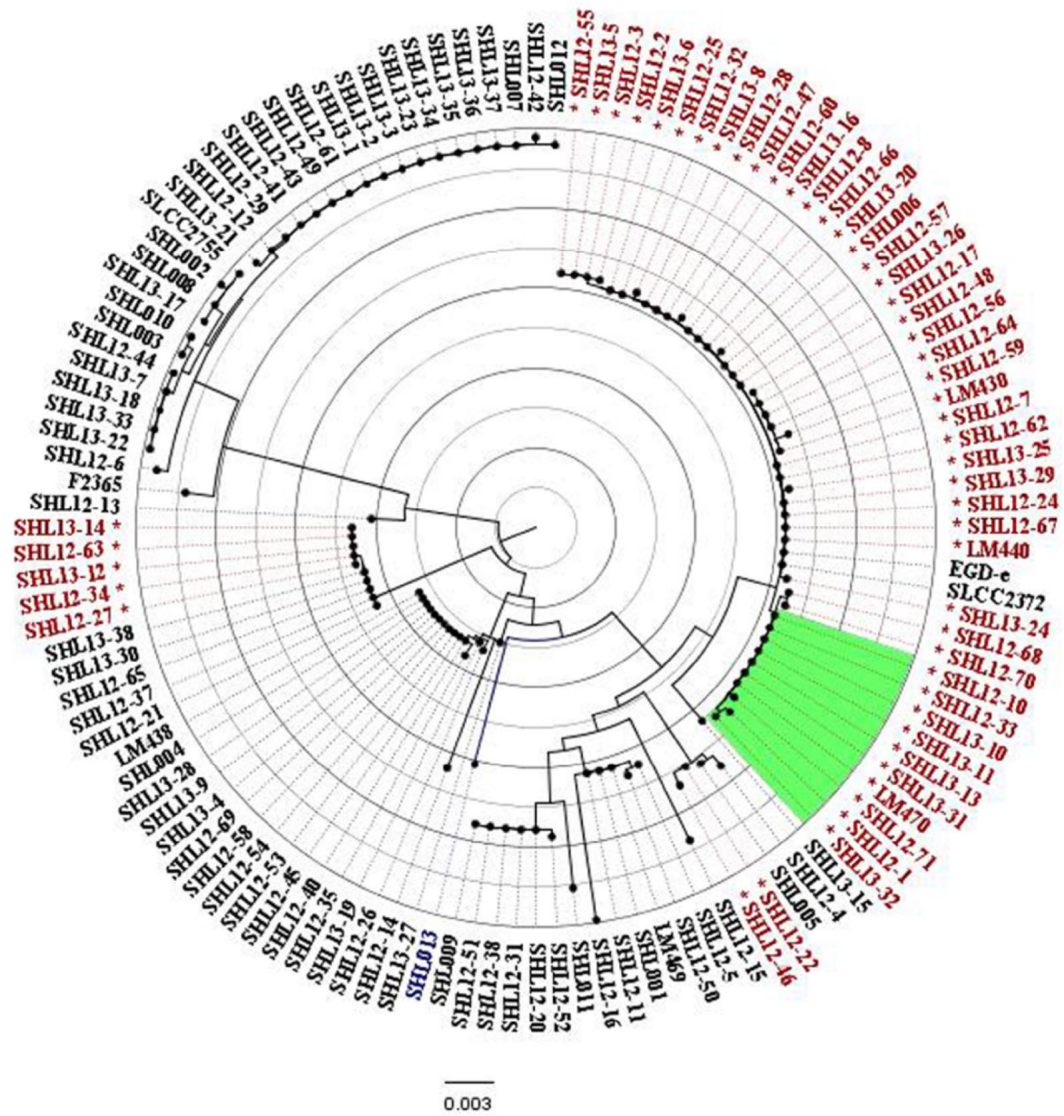
Phylogenetic tree of 120 *L. monocytogenes* isolates based on 2400 bp nucleotide sequence of *inlA*. “*” denotes isolates with PMSC mutations in *inlA.* Ten isolates with mutation type 19 in *inlA* were grouped together in the region highlighted in green

Phylogenetic analysis revealed a clear separation of *inlA* sequences between *L. monocytogenes* lineages I and II (Fig. 1). One branch contained all of the 29 isolates of serotypes 1/2b and 3b (Lineage I). Another branch contained 1/2a and 3a (except isolate SHL12-13), and 1/2c and 3c isolates (Lineage II). The clustering of the *inlA* sequences containing PMSC mutations was evident and consistent with the lineage classification of the isolates (Fig. 1). Most isolates of serogroup 1/2c, 3c with nonsense mutations were grouped together. Ten isolates with mutation type 19 in *inlA* were grouped together in the region highlighted in green in Fig. 1.

### Phylogenetic analysis of *L. monocytogenes* genomes

The genome of four *L. monocytogenes* isolates from the current was assembled, including serotype 1/2c strain SHL12-2 (16 contigs, 3.01 Mb, 583,457 bp N50 contig size, and 2978 identified genes), serotype 1/2a strain SHL12-22 (20 contigs, 3.02 Mb, 506,776 bp N50 contig size, and 2943 identified genes), serotype 1/2a strain SHL13-12 (17 contigs, 2.99 Mb, 524,215 bp N50 contig size, and 2884 identified genes), and serotype 1/2b strain SHL003 (22 contigs, 3.00 Mb, 476,214 bp N50 contig size, and 2964 identified genes). These four draft genome sequences have been deposited in GenBank under the accession numbers LRTW00000000, LRTX00000000, LRTY00000000, and LVYC00000000.

**Fig. 2:**
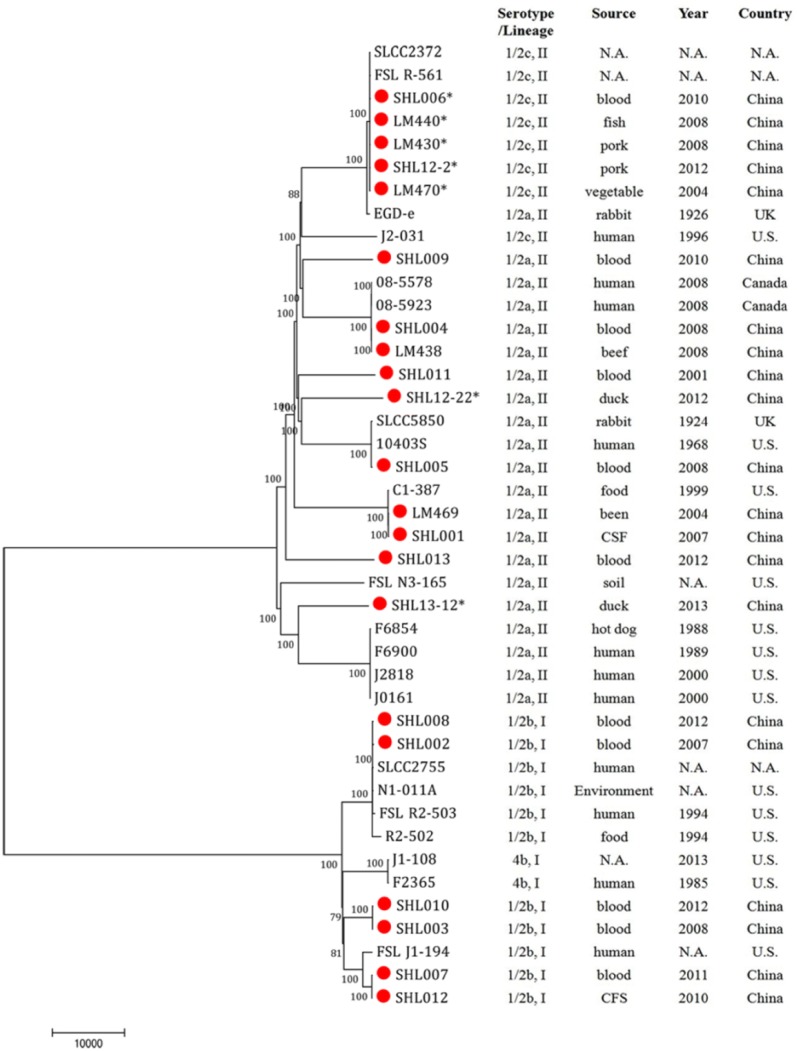
Phylogenetic tree of 42 *L. monocytogenes* genomes. A total of 153,382 SNPs in 2247 core genes were identified. The 21 *L. monocytogenes* genomes from China (our current and previous studies) are identified in red. PMSC mutations are marked with “*”

Additional 38 genomes were included for the phylogenetic analysis, resulting in 2247 core genes shared by the 42 genomes, and 153,382 SNPs. The 42 *L. monocytogenes* genomes were divided into two lineages based on the phylogenetic tree (Fig. 2). Serotype1/2c isolates carrying PMSCs in *inlA* were grouped together with nearly identical core genes sequences with SNPs ranging from 62 to 133. SHL006, LM430, LM440, LM470 and SHL12-2 with different *inlA* mutation types (types 12, 11, 4 and 19, respectively) were also grouped together with the range of 56–109 SNPs. Human isolate SHL006 with PMSC in *inlA* was grouped together with two isolates with full length *inlA*, only 134 SNPs difference between them. SHL12-22 with *inlA* mutation type 4 and SHL13-12 with *inlA* mutation type 6 were grouped into different subgroups; SHL12-22 displayed 27,265 SNP differences from SHL13-12. Both of these 1/2a isolates also retained divergent distances from 1/2c isolates that carried PMSCs. Although SHL12-22 and LM 440 carried the same mutation type in *inlA* (type 4), there was a difference of 21,981 SNPs between the core genes in their genomes.

### Invasion of Caco-2 cells by *L. monocytogenes*

**Fig. 3:**
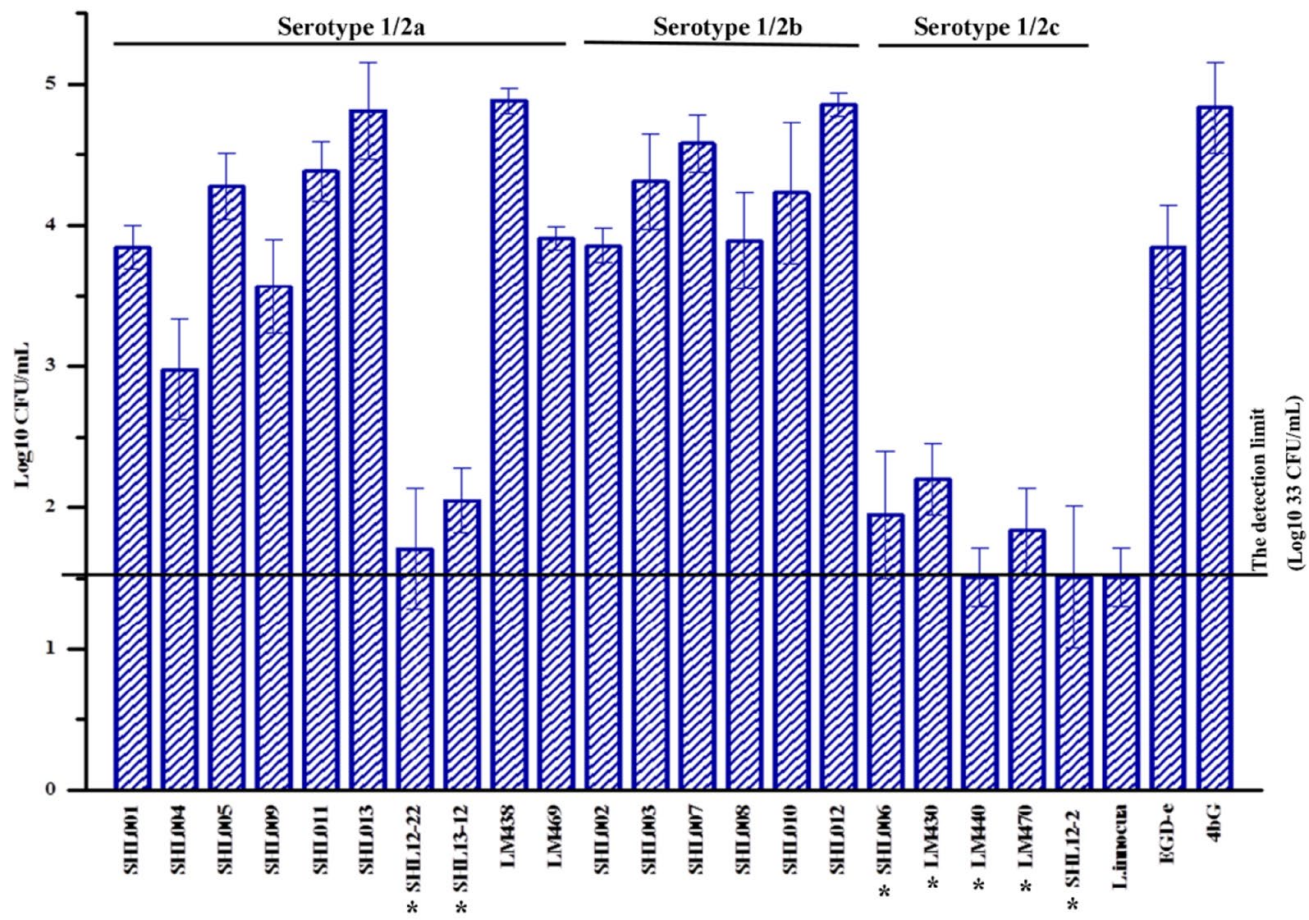
The ability of 21 *L. monocytogenes* isolates to invade human Caco-2 epithelial cells in vitro. Isolates with *inlA* PMSC mutations are marked with “*”. A black square indicates *inlA* positive

The 21 *L. monocytogenes* isolates that were tested for their ability to invade human Caco-2 cells, included: SHL006 with *inlA* mutation type 12; LM430 with mutation type 11; LM440 with mutation type 4; LM470 with mutation type 19; SHL12-2 with mutation type 8; SHL12-22 with mutation type 4, SHL13-12 with mutation type 6 (Additional file 1: Table S1), and 14 isolates with full-lenghth *inlA*. Results of the invasion assay are presented in Fig. 3 and reported as log10 CFU/mL ± standard deviation. A significant difference (*P *< 0.01) in the means of the bacterial count was observed between isolates with full-length and truncated *inl*A profiles. Serotype 1/2b isolates showed high invasion efficiencies (4.28 ± 0.39 (Log10 CFU/mL)). Two food isolates (LM440 and SHL12-2) with PCMS mutations in *inlA*, and *L. innocua* ATCC33090 failed to invade Caco-2 human epithelial cells. Food isolate LM470, that carried PCMS mutation type 19, exhibited lower bacterial counts (1.84 ± 0.30 (Log10 CFU/mL)). A correlation between the existence of PMSC mutations in *inlA* and weak Caco-2 cell invading ability was found among the isolates examined (Pearson’s coefficient 0.927, *P *< 0.01). But the internalin profiles were not associated with their ability to invade human Caco-2 cells.

Among the clinical isolates, SHL006 (obtained from a human blood sample) exhibited a lower mean of the bacterial count, 1.95 ± 0.45 (Log10 CFU/mL). The bacterial count from the isolates with full-length *inlA* that caused death in Shanghai was greater than those with PMSC mutations. However, SHL013 with a deletion in *inlA* recovered from cerebrospinal fluid showed high invasion efficiencies (4.81 ± 0.34 (Log10 CFU/mL)). SHL001 from cerebrospinal fluid also exhibited a higher bacterial count (3.85 ± 0.15 (Log10 CFU/mL)).

## Discussion

*Listeria monocytogenes* is an important foodborne pathogen with a significant public health concern worldwide. The pathogen is able to invade a number of nonphagocytic cells. The invasion efficiency of *L. monocytogenes* varies with cell lines. Entry into cells has been investigated in great detail since the discovery of invasion proteins internalin (InlA) and InlB, and their respective receptors E-cadherin (E-cad) and Met [9]. For instance, InlC may play a role when bacteria are intracytosolic, in the process of active intercellular spread [25]. Individual inactivation of internalins from the *inlGHE* gene cluster appears to modulate Listeria InlB dependent invasion of endothelial cells [2]. An *inlJ* deletion mutant significantly attenuated virulence after intravenous infections of Balb/c mice or oral inoculation of hEcad mice [45]. Drolia et al. show that *L. monocytogenes* employs Listeria adhesion protein (LAP) to exploit epithelial innate defenses and induce intestinal barrier dysfunction [12, 13]. Meanwhile, the pathogen may benefit from synergetic cooperation of different factors (Ami, p60, ActA, Auto) in invasion [5, 33, 42, 53, 56].

Virulence potential is known to vary among different strains of *L. monocytogenes* [6, 11, 27, 36]. Multiple distinct mutations leading to PMSC in inlA have previously been reported [26, 32, 36]. To date, 21 mutation types of *inlA* have been found [19, 20, 58]. Isolates carrying a PMSC mutation in inlA produce a truncated form of InlA that is secreted rather than anchored to the bacterial cell wall [37]. Food-derived isolates with PMSC mutations have been reported at similar frequency levels to those reported in the present study (30–45%) [48], and PMSC mutations in 1/2c isolates were found to be more common. Among the serotype 1/2c isolates obtained from foods, we found that 43 (93.48%, n = 46) carried PMSC mutations in *inlA*. Kanki et al. also reported that 1/2c isolates from food all carried PMSCs in Japan [26]. Two other studies have provided initial evidence that *L. monocytogenes* known to carry *inlA* PMSC mutations rarely cause human listeriosis in the United States [22, 37]. Furthermore, Van Stelten et al. have reported that a significantly (*P *< 0.001) greater proportion of RTE food isolates (45.0%) carried a PMSC mutation in *inlA* than human clinical isolates (5.1%) in the United States [58]. Genetic changes leading to a PMSC mutation in *inlA* therefore represent a molecular marker for *L. monocytogenes* virulence attenuation [26], which could therefore be used to predict the human health risk associated with consumption of food contaminated by *L. monocytogenes.*

The majority of virulence genes have been detected in the WGS data, providing a fast and cheaper alternative to conventional typing techniques [30]. In this study, we compared core genes in the 42 genomes of *L. monocytogenes* from diverse sources and regions, and found that some of these genomes carried different PMSCs in *inlA*. Serotype 1/2c showed a clonal structure, and had identical characteristics in their genomes even if they had different mutation types of *inlA*. In contrast, *L. monocytogenes* serotypes 1/2a with PMSCs in *inlA* displayed a divergent structure. Mutations of *inlA* were in fact random. Comparative sequence analysis revealed differences between the human and food isolates. Overall, we found that *L. monocytogenes* from China contained extensive diversification, consistent with the findings of Zhang et al. [62].

Many investigators have used cell culture assays to determine differences in virulence among strains of *L. monocytogenes*. In this study, the invasion ability of 13 human and 8 food isolates of *L. monocytogenes* exhibiting PMSC mutations was evaluated via a Caco-2 cell invasion assay. *L. monocytogenes* isolates with PMSCs showed reduced invasion efficiencies. A significant correlation between the existence of PMSC mutations in *inlA* and a weakness in the ability of *L. monocytogenes* isolates to invade Caco-2 cells was observed. This finding is consistent with those of several previous studies in France, Japan and the United State, reporting that *L. monocytogenes* isolates with PMSC mutations displayed reduced invasion of Caco-2 cells in vitro [26, 36, 54]. Such isolates have also been shown to exhibit low virulence levels in mammalian hosts [36]. It appears that serotype 1/2c isolates found in foods invariably carry a high rate of PMSC mutations, but the frequency in those from human cases is lower [32].

*Listeria monocytogenes* from China have recently been shown to display a divergent population structure [62]. In the present study, an isolate derived from a vegetable source (LM470) which carried PMSC mutation type 19, showed reduced invasion ability into human Caco-2 epithelial cells. Meanwhile, the other nine isolates carrying the same PCMS mutations were from chicken, pork and beef, respectively. InlA mutation isolates had a widespread distribution and we also noticed *L. monocytogenes* isolates with PMSCs might still cause listeriosis (human isolate SHL006 carrying a PMSC mutation), which have been reported in previous studies [17, 21, 31, 58]. It is also important to note that *L. monocytogenes* infection is related to additional factors such as dietary intake, and health condition (pregnant women and the elderly) [24].

## Conclusions

Our results in this study have revealed that a proportion of *L. monocytogenes* isolates from food and humans in Shanghai, China, exhibited a reduced ability to invade human intestinal epithelial cells in vitro, and that this is linked to the presence of PMSC mutations in *inlA*. The PMSC mutations are commonly carried by serotype 1/2c isolates that had identical characteristics in their genomes and were less prevalent among human *L. monocytogenes*. In agreement with previously published studies [26, 36, 39, 57], *L. monocytogenes* carrying PMSC mutations showed reduced invasion ability and attenuated virulence. It is correlated with the distribution of *L. monocytogenes* in food and humans. However, isolates with PMSC in *inlA* have been associated with human illness, and pose a risk to cause foodborne listeriosis due to their high prevalence in food. A better understanding of relationship between genetics and virulence in *L. monocytogenes* and their association with *L. monocytogenes* invasion can provide tools to develop more effective diagnostic strategies in reducing risk of human listeriosis.

## Additional file

**Additional file 1: Table S1.** Characterization of specific alleles in InlA of 120 Listeria monocytogenes isolates.

## Data Availability

These four draft genomes have been deposited in GenBank under the accession numbers: SHL003, LVYC00000000; SHL12-2, LRTW00000000; SHL12-22, LRTX00000000; SHL13-12L, RTY00000000.
